# Monitoring and control of the release of soluble O_2_ from H_2_O_2_ inside porous enzyme carrier for O_2_ supply to an immobilized d‐amino acid oxidase

**DOI:** 10.1002/bit.28130

**Published:** 2022-05-16

**Authors:** Sabine Schelch, Juan M. Bolivar, Bernd Nidetzky

**Affiliations:** ^1^ Austrian Centre of Industrial Biotechnology Graz Austria; ^2^ Institute of Biotechnology and Biochemical Engineering, NAWI Graz Graz University of Technology Graz Austria

**Keywords:** bubble‐free O_2_ supply, co‐immobilized oxidase and catalase, hydrogen peroxide, inside particle O_2_ monitoring, optical sensing, spatiotemporally controlled O_2_ release

## Abstract

While O_2_ substrate for bio‐transformations in bulk liquid is routinely provided from entrained air or O_2_ gas, tailored solutions of O_2_ supply are required when the bio‐catalysis happens spatially confined to the microstructure of a solid support. Release of soluble O_2_ from H_2_O_2_ by catalase is promising, but spatiotemporal control of the process is challenging to achieve. Here, we show monitoring and control by optical sensing within a porous carrier of the soluble O_2_ formed by an immobilized catalase upon feeding of H_2_O_2_. The internally released O_2_ is used to drive the reaction of d‐amino acid oxidase (oxidation of d‐methionine) that is co‐immobilized with the catalase in the same carrier. The H_2_O_2_ is supplied in portions at properly timed intervals, or continuously at controlled flow rate, to balance the O_2_ production and consumption inside the carrier so as to maintain the internal O_2_ concentration in the range of 100–500 µM. Thus, enzyme inactivation by excess H_2_O_2_ is prevented and gas formation from the released O_2_ is avoided at the same time. The reaction rate of the co‐immobilized enzyme preparation is shown to depend linearly on the internal O_2_ concentration up to the air‐saturated level. Conversions at a 200 ml scale using varied H_2_O_2_ feed rate (0.04–0.18 mmol/min) give the equivalent production rate from d‐methionine (200 mM) and achieve rate enhancement by ∼1.55‐fold compared to the same oxidase reaction under bubble aeration. Collectively, these results show an integrated strategy of biomolecular engineering for tightly controlled supply of O_2_ substrate from H_2_O_2_ into carrier‐immobilized enzymes. By addressing limitations of O_2_ supply via gas‐liquid transfer, especially at the microscale, this can be generally useful to develop specialized process strategies for O_2_‐dependent biocatalytic reactions.

## Graphical abstract

Supply of O_2_ substrate to biotransformations in porous carriers requires tailored solutions beyond bubble aeration. Here, the authors used immobilized catalase (CAT) to produce O_2_ from H_2_O_2_ inside the carrier to generate O_2_ for reaction of a co‐immobilized D‐amino acid oxidase (DAAO). O_2_ measurement in liquid and inside particle (based on a co‐immobilized luminescence dye) guided development of a H_2_O_2_ feeding strategy for tightly controlled O_2_ supply. This enabled rate enhancement compared to the same oxidase reaction under bubble aeration.

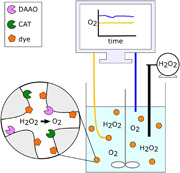

**Abbreviations**
CAT: catalase (from *Bordetella pertussis*)
DAAO: d‐amino acid oxidase (from *Trigonopsis variabilis*)
Z‐CAT and Z‐DAAO: CAT and DAAO harboring the cationic binding module Z_basic2_ fused to the N‐terminus of the enzyme

## 1 INTRODUCTION

**Figure 1:**
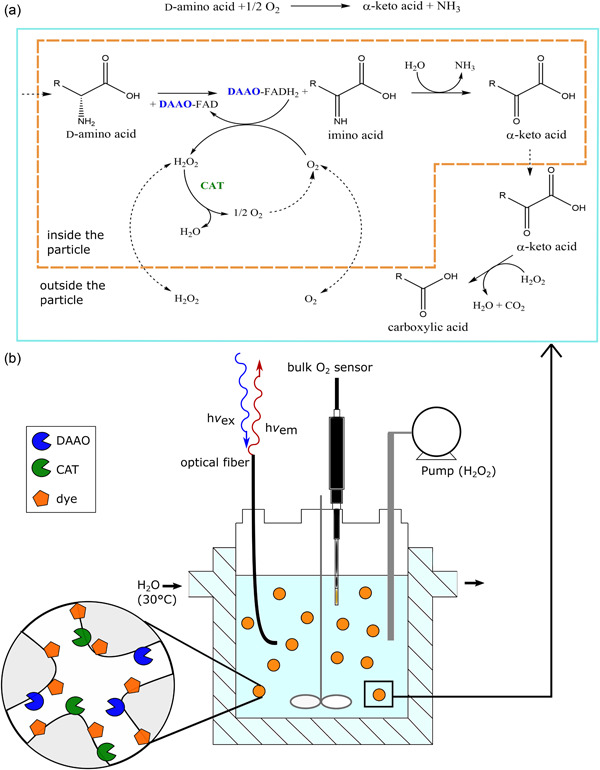
Strategy of controlled O_2_ supply from H_2_O_2_ to immobilized DAAO in the presence of co‐immobilized CAT. (a) Net chemical reaction catalyzed by CAT and DAAO (*top*); detailed reaction scheme within the particle and diffusion of substrates into and out of the particle (dashed arrows), the off‐pathway chemical decarboxylation of the α‐keto acid product by H_2_O_2_ occurs outside of the particle, where no CAT is present (*bottom*). (b) Schematic of the reactor set‐up used for controlled supply of H_2_O_2_. The labeled co‐immobilizate is shown as orange circles. Bulk oxygen sensor and optical fiber (*h*ν_ex_, excitation; *h*ν_em_, emission) is used for measuring external and internal (inside the particle) O_2_, respectively. DAAO, d‐amino acid oxidase; CAT, catalase.

Enzyme‐catalyzed O_2_‐dependent oxidation reactions have considerable importance in organic synthesis, carried out up to the industrial manufacturing scale (Dong et al., 2018; Puetz et al., 2020; Romero et al., 2018; Winkler et al., 2021; Wu et al., 2021). Fundamental problem of biochemical engineering of these reactions is to supply the O_2_ substrate in a way that meets the demands of conversion efficiency and enzyme stability, both in suitable balance (Bolivar et al., 2019; Dong et al., 2018; Toftgaard Pedersen et al., 2015; Woodley, 2019). Commonly, the O_2_ is delivered from gas (air, pure oxygen) entrained into the bulk liquid (Bolivar et al., 2019; Garcia‐Ochoa & Gomez, 2009; Lindeque & Woodley, 2020; Solé et al., 2019; Thomas et al., 2021; Tomaszewski et al., 2014; Van Hecke et al., 2011). Irrespective of type and scale of the apparatus used for the reaction, efficient gas‐liquid contacting requires some form of two‐phase fluid flow (e.g., turbulent flow in agitated tanks; dispersed‐phase or separated‐phase laminar flow in pipes or channels; Birmingham et al., 2021; Solé et al., 2019; Tomaszewski et al., 2014; Van Hecke et al., 2011; Zverina et al., 2021). However, the O_2_ supply from gas‐liquid contacting confronts severe limitations when fluids become effectively stagnant. An important case of stagnant fluid is the liquid filling the macro‐pores (≤100 nm width) of solid particles used as carriers for enzyme immobilization (Bolivar & Nidetzky, 2019; Bolivar et al., 2013; Bolivar, Eisl, et al., 2016; Lorente‐Arevalo et al., 2021b). To achieve enhancement of the O_2_ supply into such solid bio‐catalysts (i.e., the stagnant aqueous fluid in them), there are essentially two options. One is to increase by partial pressure the O_2_ concentration in the bulk liquid and in that way enhance the O_2_ transfer rate by pore diffusion (Bolivar et al., 2019; Toftgaard Pedersen et al., 2015). The other is to chemically generate O_2_ in the stagnant fluid (Chapman et al., 2018; Schneider et al., 2012; Van Hecke et al., 2009; Yoshimoto & Higa, 2014). A promising strategy of localized release of O_2_ involves H_2_O_2_ supplied in the bulk liquid and soluble O_2_ formed by a catalase (H_2_O_2_ → ½ O_2_ + H_2_O) immobilized in the solid carrier (Bolivar, Schelch, et al., 2016). Immobilized, catalase‐like chemical catalysts could likewise be employed (Bao et al., 2004; Schussel & Atwater, 1996; Vikartovska‐Welwardova et al., 1999). The two options have complementary uses in reaction engineering. Reaction at high pressure offers unique opportunities for process intensification of O_2_ dependent enzymatic conversions in continuous flow, as shown by some of the authors elsewhere (Bolivar et al., 2019). Localized release of O_2_ from externally added H_2_O_2_ is particularly useful in miniaturized reaction systems (e.g., microscale analytical devices and reactors) in which defined gas‐liquid contacting can be complicated technically and spatiotemporal control over the available O_2_ is desired (Bolivar & Nidetzky, 2013; Chapman et al., 2018; Cosgrove et al., 2019; Mattey et al., 2021). Here, we focused on this latter strategy with the aim of demonstrating controlled in‐situ supply of the O_2_ substrate into carrier‐bound solid preparation of an immobilized O_2_‐dependent enzyme (Figure 1).

The current study builds on advance from Bolivar et al. (2016) in two ways. First, the catalase (CAT) from *Bordetella pertussis* was designed for facile immobilization. The enzyme was fused with the cationic binding module Z_basic2_ at its N‐terminus. The Z‐CAT fusion protein was adsorbed on ReliSorb SP400 (a polymethacrylate carrier with anionic sulfonate surface groups) with high affinity and selectivity. The immobilized Z‐CAT retained up to ∼80% of the specific activity of the soluble enzyme. Second, the ReliSorb SP400 carrier with Z‐CAT immobilized on it was labeled with a Ru(II) luminophore for optical sensing of O_2_. Time‐resolved measurements from inside the solid carrier were used to monitor the O_2_ released from H_2_O_2_ by the immobilized Z‐CAT. When H_2_O_2_ was supplied, a strongly positive O_2_ concentration gradient between the carrier (high [O_2_]) and the surrounding bulk liquid (lower [O_2_]) was developed. In the presence of an O_2_ consuming reaction in solution (d‐glucose oxidation by glucose oxidase), the gradient of [O_2_] (Δ[O_2_]) reached values that exceeded by up to ∼1.5‐fold the air‐saturated equilibrium O_2_ concentration in solution at atmospheric pressure. Note: provision of O_2_ substrate in the bulk liquid through gassing necessarily results in an O_2_ concentration gradient of *opposite* (negative) sign when the O_2_ consuming enzymatic reaction happens in the solid carrier. The ratio of the locally available [O_2_] and the enzyme *K*_m_ for O_2_ determines the degree to which the rate of the reaction catalyzed by the immobilized enzyme depends on the O_2_ concentration (for a general discussion of the O_2_ *K*_m_ related to enzyme conversion efficiency, see Bolivar et al., 2019; and Toftgaard Pedersen et al., 2015). Therefore, these results suggested the exciting possibility of a tunable regulation of the immobilized enzyme activity, made possible through the spatiotemporally controlled, and tightly monitored, supply of O_2_ from the added H_2_O_2_. The current study was performed to demonstrate applicability of the integrated process concept together with its associated process analytical technology. The O_2_ dependent reaction of an immobilized d‐amino acid oxidase (DAAO; from the yeast *Trigonopsis variabilis*) was examined (Figure 1a). The DAAO in immobilized form is well known for its application in large‐scale industrial bio‐catalysis (conversion of cephalosporin C; Gröger et al., 2017; Volpato et al., 2010). It is also of considerable interest for bio‐sensor development (e.g., measurement of the brain metabolite d‐serine; Moussa et al., 2021). Due to its high *K*_m_ for O_2_ (∼1 mM; Pollegioni et al., 1993), the DAAO presented an interesting example to show the effect of internal supply of O_2_. Under the operating conditions considered for the current study (air; atmospheric pressure), the degree of use of the available oxidase activity shows effectively linear dependence on the carrier‐internal O_2_ concentration.

## 2 MATERIALS AND METHODS

### 2.1 Materials, enzymes, and assays

Dichloride (4,7‐diphenyl‐1,10‐phenanthroline) ruthenium (II), Ru(dpp)_3_, was from ABCR GmbH. Chemicals were of analytical grade from Sigma‐Aldrich. ReliSorb SP400 carrier was a kind gift from Resindion. As stated by the supplier, the carrier is porous (pore diameter: 40–50 nm) and spherical (particle diameter: 75–200 µm). Horseradish peroxidase and *Aspergillus niger* d‐glucose oxidase (Type II‐S, 15,000–50,000 units/g solid) were from Sigma‐Aldrich.

Z‐DAAO (from *T. variabilis*) and Z‐CAT (from *B. pertussis*) were produced in *Escherichia coli* BL21 (DE3) as described by Wiesbauer et al. (2011) and Bolivar et al. (2016), respectively.

Roti®Quant protein assay (Carl Roth) was used with BSA as reference. Activity of Z‐CAT (soluble, immobilized, co‐immobilized with Z‐DAAO) was determined (30°C, pH 8.0, 50 mM potassium phosphate buffer) by measuring the decrease in absorbance of H_2_O_2_ (20 mM) at 240 nm in a stirred quartz cuvette (Betancor et al., 2003). Activity of Z‐DAAO (soluble, immobilized, and co‐immobilized with Z‐CAT) was determined (30°C, pH 8.0, 50 mM potassium phosphate buffer) with a coupled peroxidase assay (Bolivar et al., 2014). Alternatively, it was determined directly by measuring the O_2_ consumption with a fiber‐optic microsensor (Bolivar et al., 2014). The substrate was d‐Met (10 mM). O_2_ was available from air‐saturated substrate solution. One unit of activity is the enzyme amount for 1 µmol/min substrate consumed or product released under the conditions used.

### 2.2 Enzyme (co)‐immobilization

Enzyme in *E. coli* lysate (100–300 µl; Z‐DAAO: 20–80 U/ml; Z‐CAT: 1–10 × 10^3^ U/ml) was diluted into buffer (50 mM potassium phosphate buffer, pH 7.0) containing 0.25 M NaCl. The total volume (1 ml) was incubated with 100 mg (dry mass) ReliSorb SP400 for 1 h in an end‐over‐end rotator at ~22°C (room temperature). Solid immobilizate was recovered. The supernatant was used to determine residual soluble protein and activity (measured at air saturation). The immobilizate was washed once with immobilization buffer and stored at 4°C in the same buffer lacking NaCl. Enzymes were co‐immobilized by incubating Z‐DAAO and Z‐CAT together at once or sequentially in varied order. Sequential immobilization involved washing of the carrier two times with buffer (1 ml) before adding the second enzyme.

The immobilization yield (*Y*_*A*_, %) was determined from the activity balance in the supernatant according to Equation (1). *A*_0_ is the initial volumetric activity (U/ml) in the loading buffer and *A*_*L*_ is the volumetric activity in the supernatant remaining after the immobilization. Both activities were measured at air saturation. Control experiments performed in the absence of carrier showed that *A*_0_ of both Z‐DAAO and Z‐CAT was stable during the time of the immobilization.

(1) $$Y_{A}=100\%\times \left(A_{0}–A_{L}\right)/A_{0}$$

The observable activity at air saturation of the immobilized enzyme preparation (*a*_*I*_) was measured directly using the activity assays described in Section 2.1. It is expressed as a specific activity in U/g dry carrier.

The effectiveness factor (*η*) compares the observable and theoretical activity of the immobilized enzyme preparation according to Equation (2). *a*_T_ is obtained as (*A*_0_−*A*_L_)/g dry carrier. Note the importance of a stable enzyme to determine *a*_T_.

(2) $$\eta =100\%\times a_{I}/a_{T}$$

### 2.3 Measurement of O_2_ in solid carrier

ReliSorb SP400 with enzymes co‐immobilized in it was used. The O_2_ concentration in the bulk solution was measured with an ultrahigh speed fiber‐optic oxygen microsensor (PyroScience GmbH) connected to a fiber‐optic oxygen meter (model FireSting®‐O_2_ from PyroScience GmbH; Bolivar et al., 2013).

Enzymatic reactions were done in an open glass vial (1.2 cm diameter) at atmospheric pressure (~250 µM [O_2_] at 30°C; air saturation) with a working volume of 4 ml (50 mM potassium phosphate buffer, pH 8.0). The vial was placed in a water bath (30°C) and magnetic stirring (6 × 3 mm^2^; 300 rpm) was used for mixing. Unless otherwise mentioned, the carrier concentration used in the reported experiments was 5.0 mg/ml.

The luminescence dye Ru(dpp)_3_ (2.5 mg; in 5% [v/v] of ethanol [98%]) was added to buffer‐soaked carrier (~1.0 g), incubated for 60 min and washed with buffer. The Ru(dpp)_3_ was stably absorbed by the carrier. The general setup for measuring O_2_ inside the carrier was described in Bolivar et al. (2016). Briefly, the luminescence lifetime of the Ru(dpp)_3_ is quenched dynamically by the O_2_ present and the phase modulation technique is used to determine the lifetime. The phase was measured with a 2‐mm fiber‐optic cable that was suitably interfaced with the reaction vessel, as shown in Bolivar et al. (2013), and connected to a fiber‐optic oxygen meter (model pH‐1 mini, PreSens–Precision Sensing GmbH). The phase shift response was calibrated by simultaneous measurement of the bulk O_2_ concentration with the microsensor at different O_2_ concentrations (10–920 µM). The experiment in Supporting Information: Figure S1 shows the calibration. The bulk [O_2_] in a solution of 100 mM glucose was brought to its maximum value by bubbling with pure O_2_ gas. The O_2_ consumption was then started by adding glucose oxidase to give a final activity of 5 U/ml. The decrease in the bulk [O_2_] measured with the microsensor was correlated with the phase shift change (Supporting Information: Figure S1b).

The manufacturer‐stated response time of the bulk microsensor for measurement of aqueous samples was 0.3 s or less. The dynamic response of the particle O_2_ sensor was identical to that of the bulk microsensor in the calibrated range of 10 µM to ~1.0 mM (solubility limit of O_2_ at ambient pressure), as shown in earlier studies (Bolivar et al., 2014, 2015, 2016). The response time of the O_2_ particle sensor was shown to be suitable for the measurements intended. Using varied amounts of glucose oxidase in the presence of glucose (100 mM), an O_2_ consumption rate between 0.15 and 50 µM/s was established. The time courses, hence the rates, recorded with the O_2_ particle sensor were identical to those recorded with the fiber‐optic microsensor. Absence of delay in the response of the particle sensor was thus confirmed for the relevant range of reaction rates.

Periodic additions of H_2_O_2_ were made from a concentrated stock solution (200 mM) in 50 mM potassium phosphate buffer, pH 8.0. The added volume was adjusted (25–100 µl) to supply the desired amount of H_2_O_2_ (5.0–20.5 µmol), resulting in concentration changes between 1.25 and 5.0 mM. The total volume change from several additions (≤10) was ~10% for most experiments. A few initial experiments were performed at higher additions of H_2_O_2_ (Δ[H_2_O_2_] = +5 mM), here the volume change was ~25%. The reported results are corrected of the volume change.

### 2.4 Progress curve analysis

**Figure 2:**
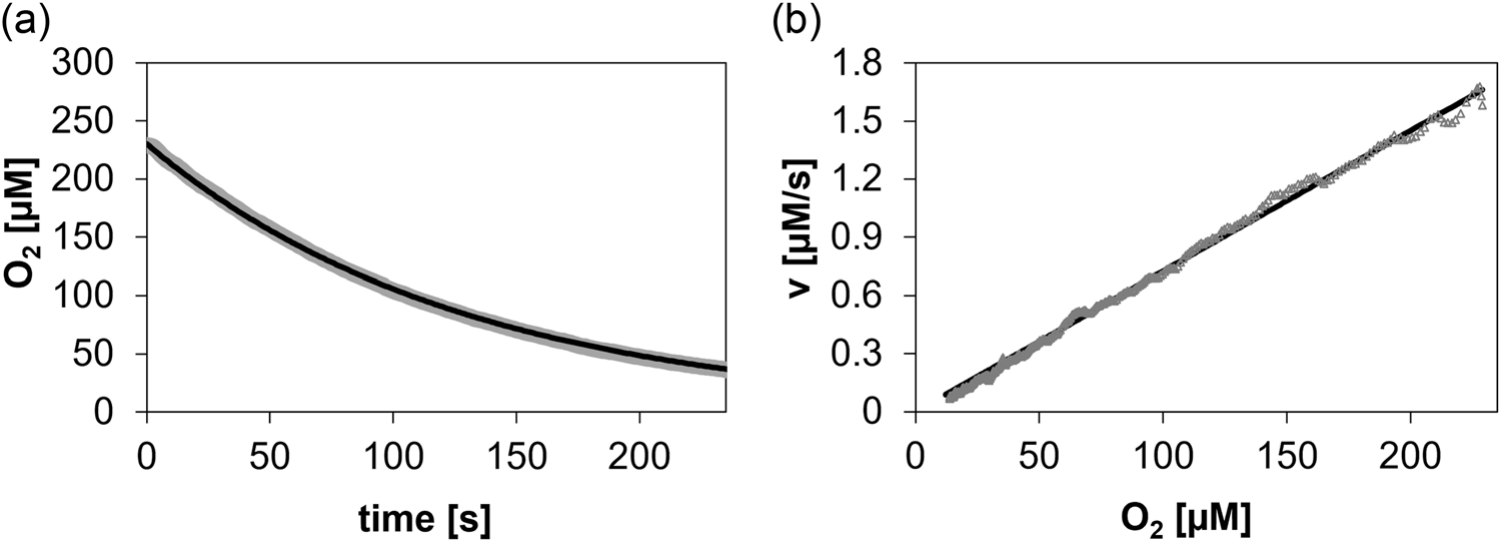
Progress curve analysis to determine dependence of the enzymatic rate on the O_2_ concentration. (a) Experimental time course (gray), and associated nonlinear fit (black), of O_2_ consumption during reaction of enzyme co‐immobilizate (Z‐DAAO: 600 U/g carrier; Z‐CAT: 50,000 U/g carrier; 5.0 mg carrier/ml) with 10 mM d‐Met as substrate. The fit is with Equation (3b). (b) Dependence of the reaction rate, obtained by differentiation of the time course in panel (a), on the O_2_ concentration. DAAO, d‐amino acid oxidase; CAT, catalase.

Reaction time courses were recorded by continuous online measurement of the [O_2_] in the bulk solution. Immobilized Z‐DAAO was used and the d‐Met substrate was saturating (10 mM; *K*_m_ = ∼0.29 mM; Schräder & Andreesen, 1996). O_2_ was available from air‐saturated substrate solution. For integral analysis of the progress curves of [O_2_] decrease (Figure 2a), direct fitting of Equation (3a) or (3b) with Berkeley Madonna™(version 10.2.8) was used. Euler's method was used as a fixed‐step integration method and the function Curve Fit based on the minimization of the root of mean squared errors was applied.

(3a) $$d\left[O_{2}\right]/dt=-V_{max}\left[O_{2}\right]/\left(K_{m}+\left[O_{2}\right]\right)$$

(3b) $$d[O_{2}]/dt=-k_{O_{2}}[O_{2}]$$

*K*_m_ (mM) is the apparent Michaelis constant of the immobilized Z‐DAAO and *V*_max_ is the maximum volumetric activity in mmol/(L min). The *V*_max_ results from *a*_*I*_ (U/g) times the carrier concentration used (g/L). *k*_O2_ is a first‐order rate constant (min^−1^), formally equivalent to the apparent efficiency of the enzyme preparation (*V*_max_/*K*_m_).

Differentiation of progress curves was done in OriginPro 2019 (version 9.6.0.172) using the function Differentiate (Savitzky‐Golay Smooth algorithm). The time interval was 20 s and the mean [O_2_] from the interval was used. Reaction rates dependent on [O_2_] are thus calculated (Lorente‐Arevalo et al., 2021a, 2022).

### 2.5 Enzyme reactor for O_2_ supply via controlled feeding of H_2_O_2_

A jacketed glass reactor (diameter: 5 cm; height: 11 cm) with a working volume of ∼200 ml was used. It was connected to an external water bath for temperature control (30°C). As shown in Figure 1b, the reactor had openings on its top that were used to fit the bulk oxygen sensor and the fiber optic cable and through which H_2_O_2_ solution (50 mM potassium phosphate buffer, pH 8.0) was fed with a Knauer model Smartline 100 HPLC pump (flow rate: 0.1–1.0 ml/min; ±10%). The concentration of labeled carrier with co‐immobilized enzymes was 5.0–15 mg/ml. A magnetic stirrer (length: 3 cm; 300 rpm) was used for mixing. Using visual inspection and analysis with the light microscope, particles were confirmed to be suitably stable over the time of the experiment (≤4 h) under stirring.

Reaction was started by adding the d‐Met substrate (200 mM) when the recorded sensor signals were stable. Once nearly all of the O_2_ in bulk liquid had been consumed, the H_2_O_2_ feed was switched on (stock solution: 100 mM H_2_O_2_). The feed rate was increased gradually every 2–3 min from the start value of 0.1 ml/min until a maximum value of 1.0 ml/min was reached. Samples (1 ml) were taken before the H_2_O_2_ addition and afterwards every 15–20 min up to ∼4 h. The total volume change from the H_2_O_2_ feed was up to ∼42%. The reported results are properly corrected for the volume change. In a separate set of experiments, the conversion of d‐Met was studied at different constant rates of H_2_O_2_ addition between 0.035 and 0.185 mmol/min.

Additional experiment involved O_2_ supply by bubble aeration. The conditions of reactor operation were identical, except that air was flown into the liquid through a Teflon microtube (inner diameter: 5 mm) equipped with a microsparger at the end. The air flow was regulated manually to adjust an external [O_2_] of 150 µM (±15%).

### 2.6 Analytics

Photometric assay (absorbance detection at 440 nm) was used for determination of the α‐keto‐acid product formed from d‐Met by Z‐DAAO. The assay is based on reaction of α‐keto‐acid with 2,4‐dinitrophenylhydrazine (1.0 mM in 2 M HCl; Bolivar et al., 2015). Samples were centrifuged shortly at 7,000 rpm (Phoenix Instrument CD‐1008 mini‐centrifuge) to remove the co‐immobilizate. The supernatant was directly incubated with 2,4‐dinitrophenylhydrazine.

Reaction samples were further analyzed with ion‐pairing HPLC. For this, they were mixed with the same volume of pure acetonitrile to stop the reaction. Solid material was centrifuged off for 30 min. d‐Met and α‐keto‐γ‐(methylthio)‐butyric acid were quantitated (UV absorbance at 210 nm) using authentic reference compounds (Sigma‐Aldrich) for calibration. A Shimadzu Prominence HPLC‐UV system (Shimadzu) equipped with a Chromolith® HighResolution RP‐18 column (100 × 4.6 mm^2^; Merck Chemicals) and a UV detector (210 nm) was used. Water containing tetrabutylammonium phosphate (6 mM) as an ion‐paring reagent was used for elution (40°C) at a flow rate of 2 ml/min.

## 3 RESULTS AND DISCUSSION

### 3.1 Enzyme co‐immobilization, and enzyme carrier labeling for optical sensing

The Z‐DAAO was immobilized on ReliSorb SP400 at nearly 100% yield at a loading of 200–800 U/g carrier (Supporting Information: Table S1 lists the yield for 600 U/g carrier). Reactions (d‐Met oxidation) were performed with the immobilized enzyme preparations and change in the O_2_ concentration was monitored in the carrier (internal [O_2_]) and in solution (external [O_2_]). As shown in Supporting Information: Figure S2, the internal [O_2_] decreased faster than the external [O_2_], resulting in a temporary gradient (Δ[O_2_] = internal [O_2_]−external [O_2_]) of negative sign. For immobilized preparations involving enzyme loadings of 400 U/g carrier or higher, the Δ[O_2_] was as large as −120 µM. A fast initial depletion of the internal [O_2_] to just ∼30% of the corresponding [O_2_] available in solution at air saturation was observed (Supporting Information: Figure S2). The effectiveness factor (*η*) of the immobilized Z‐DAAO was only 8.5% (600 U/g), likely explainable by the low supply of O_2_ from the bulk liquid into the solid carrier. Previous studies of the authors (Bolivar et al., 2014, 2015) have shown rate limitation by O_2_ diffusion into immobilized preparations of the Z‐DAAO. Reactions at high enzyme loading (≥600 U/g) showed almost complete consumption of the O_2_ in the carrier and in solution within ∼150 s (Supporting Information: Figure S2, panels c,d). Immobilized preparations of Z‐DAAO giving rapid consumption of the O_2_ inside the solid carrier were particularly suitable for studying the effect of internal O_2_ supply from H_2_O_2_. Considering the possible interference from large protein loadings on labeling of the ReliSorb SP400 carrier with Ru(dpp)_3_ (Bolivar et al., 2016), we immobilized the Z‐DAAO at 600 U/g for further study.

From earlier work on the immobilization of Z‐CAT (Bolivar et al., 2016), we additionally selected to immobilize the Z‐CAT at 50,000 U/g carrier. The yield for this immobilization of Z‐CAT was ∼100% (Supporting Information: Table S1). The enzyme effectiveness was ∼7%. Co‐immobilization of Z‐DAAO and Z‐CAT was examined via simultaneous or sequential immobilization of the two enzymes. The simultaneous process is step‐economic, but it also involves the largest volumetric load of protein from the *E. coli* cell lysates (Bolivar & Nidetzky, 2012). Nonspecific protein binding was therefore considerably lower when Z‐DAAO and Z‐CAT were immobilized one after the other (data not shown). The order of enzyme immobilization had only a small influence on the activity yield and enzyme effectiveness (Supporting Information: Table S1). Compared to the results of single enzyme immobilization of Z‐DAAO and Z‐CAT, the effectiveness factors (*η*) of the two enzymes in the co‐immobilized catalyst preparation appeared to be lower by ∼50% (Supporting Information: Table S1). However, when co‐immobilized on the same carrier, the assays for the individual enzyme activity can be affected by the presence of the respective other co‐immobilized enzyme (Bolivar et al., 2017), and this may have been a reason for some decrease in the measured value of *η*. Labeling with Ru(dpp)_3_ did not change the *η* value (Supporting Information: Table S1). Considering size of the functional enzyme (Z‐CAT, homo‐tetramer, ∼260 kDa; Z‐DAAO, homo‐dimer, ∼76 kDa), the relatively larger Z‐CAT was immobilized first. We assumed that a more homogenous distribution of the two enzymes into the carrier might be achieved in that way. We obtained a co‐immobilized enzyme preparation with an activity of 4,500 (±590; *N* = 6) U Z‐CAT/g and 27 (±1.8; *N* = 6) U Z‐DAAO/g.

The labeled ReliSorb SP400 particles (2.5 mg Ru(dpp)_3_/g dry carrier), with Z‐CAT and Z‐DAAO immobilized in them, gave a suitable response to change in [O_2_] in the bulk solution. This is shown in Supporting Information: Figure S1a. The decrease in [O_2_] due to the reaction of soluble glucose oxidase was monitored with both the O_2_ microsensor and the labeled particles. Note: no reaction by the immobilized Z‐CAT and Z‐DAAO took place in this experiment. The time courses of [O_2_] recorded from signal of sensor and labeled particles were almost superimposable, thus validating the particle‐based method of [O_2_] measurement. Calibration of the method is shown in Supporting Information: Figure S1b.

Comparing the reaction of the co‐immobilized enzyme preparation with the reaction of Z‐DAAO immobilized individually, we observed a similar trend for the time‐dependent change of the internal [O_2_] relative to the [O_2_] in bulk solution. In both enzyme preparations, as shown in Supporting Information: Figure S3, the internal [O_2_] decreased considerably faster than the [O_2_] in bulk solution. Measurement of the internal [O_2_] in the co‐immobilized enzyme preparation was validated from these results. Multiple measurements showed excellent reproducibility (≤5% deviation) of the internal and external [O_2_] time courses when the same batch of co‐immobilized enzyme was used. The largest variation resulted from Z‐CAT activity (≤13%) in different preparations of co‐immobilized enzyme.

From progress curve analysis as shown in Figure 2, we determined the rate versus concentration profile for the co‐immobilized enzyme preparation. The O_2_ consumption rate was linearly dependent on the liquid bulk O_2_ concentration up to the maximum of ∼250 µM used (air saturation). This implies enzymatic reaction by first‐order kinetics with respect to [O_2_] in the relevant concentration range. In other words, the *K*_m_ of the immobilized Z‐DAAO for O_2_ (which is indeterminate from the results shown) exceeds by far the maximally used [O_2_]. This emphasizes the importance of efficient supply of O_2_ to the reaction of the immobilized Z‐DAAO.

### 3.2 Dynamics of the internal and external O_2_ concentration upon the addition of H_2_O_2_

**Figure 3:**
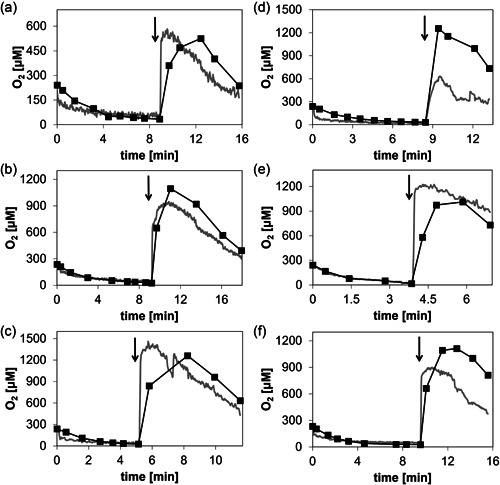
The dynamics of the internal and external O_2_ concentration upon H_2_O_2_ addition to different preparations of immobilized enzyme. (a–c) Internal release of O_2_ dependent on the amount of H_2_O_2_ added, resulting in a calculated H_2_O_2_ concentration increase of 2.50 mM (a), 3.75 mM (b), and 5.00 mM (c). The time of H_2_O_2_ addition is indicated by an arrow. Time courses of internal [O_2_] (gray line, continuous measurement) and external [O_2_] (black squares connected by line, discontinuous measurement at the indicated times) were recorded. Enzyme co‐immobilizate containing Z‐DAAO at 600 U/g carrier and Z‐CAT at 50,000 U/g carrier was used. (d–f) Comparison of O_2_ consumption and production (calculated H_2_O_2_ concentration increase after the H_2_O_2_ addition, 3.75 mM) with different combinations of free and immobilized Z‐DAAO and Z‐CAT. (d) Immobilized Z‐DAAO (600 U/g carrier) and free Z‐CAT (equivalent to 50,000 U/g carrier); (e) free Z‐DAAO (equivalent to 600 U/g carrier) and immobilized Z‐CAT (50,000 U/g carrier); and (f) co‐immobilized Z‐DAAO (600 U/g carrier) and Z‐CAT (50,000 U/g carrier). All reactions contained 10 mM d‐Met and (co)‐immobilizate was used at 5 mg/ml. For further details, see Section 2.3.

Figure 3 shows time courses of the internal and external [O_2_] recorded upon the addition of H_2_O_2_ to the enzyme co‐immobilizate at a point when the soluble O_2_ in the system had been largely consumed by the oxidation of d‐Met. Overall, both the internal and the external [O_2_] increased temporarily, passed through a maximum and decreased later again. The shape of the recorded course for the internal [O_2_] was strongly dependent on the amount of H_2_O_2_ added (Figure 3a–c). The internal [O_2_] increased almost instantaneously after the H_2_O_2_ was added. The magnitude of the burst‐like [O_2_] release increased when the [H_2_O_2_] was increased. The external [O_2_] increased only gradually, and with some delay, compared to the internal [O_2_]. The maximum [O_2_] was thus reached more slowly externally. The maximum [O_2_] levels were, however, comparable inside the carrier and in solution. The later decrease in the [O_2_] occurred at the same rate internally and externally. It was dependent on the amount of H_2_O_2_ added (Figure 3a–c). In the decline phase of the [O_2_] time course (Figure 3a), the internal [O_2_] was consistently smaller than the external [O_2_]. Diffusion of O_2_ between particle and bulk solution can probably explain these observations. Some of the O_2_ released from H_2_O_2_ might diffuse from the solid particle (e.g., outer regions of the particle) into the liquid bulk before the maximum [O_2_] is reached. In the decline phase of the [O_2_] time course, the O_2_ consumption in the particle is due to the reaction of the immobilized Z‐DAAO, while the decline of [O_2_] in the bulk is caused by the diffusion of O_2_ from bulk into the particle.

To demonstrate that the dynamics of the internal [O_2_] was dependent on the immobilized Z‐CAT activity, we prepared a second co‐immobilizate in which the immobilized Z‐DAAO activity was the same, but 10,000 U/g instead of 50,000 U/g of the Z‐CAT were immobilized. The maximum [O_2_] released internally was considerably lower (∼75 µM compared to ∼185 µM) when a smaller Z‐CAT activity was immobilized (Supporting Information: Figure S4).

To show the interplay between the co‐immobilized Z‐CAT and Z‐DAAO activities in releasing and consuming the soluble O_2_ in the heterogeneous environment of the solid carrier, we compared the H_2_O_2_‐driven reaction of the co‐immobilized enzyme preparation with the corresponding enzymatic reactions in which only Z‐CAT or Z‐DAAO was immobilized and the respective other enzyme was added as a soluble preparation (Figure 3d–f). When Z‐CAT was soluble and Z‐DAAO immobilized, the external [O_2_] increased faster, and reached a higher maximum level, than the internal [O_2_] upon the addition of H_2_O_2_ to the suspension (Figure 3d). The trend was opposite when Z‐DAAO was soluble and Z‐CAT immobilized: the internal [O_2_] increased faster and to a higher level than the external [O_2_] (Figure 3e). Finally, when Z‐DAAO and Z‐CAT were co‐immobilized, the increase in [O_2_] was faster internally, but in the absence of O_2_ consumption in external solution, a higher concentration of O_2_ accumulated there (Figure 3f). Figure 3b,f shows [O_2_] time courses for the same experimental setup with different batches of the co‐immobilizate. The individual enzyme activities varied by maximally 13% in *N* = 6 independent experiments. Slight differences in the time courses are explained by this variation of immobilized activity in different batches of co‐immobilized enzyme preparation used.

**Figure 4:**
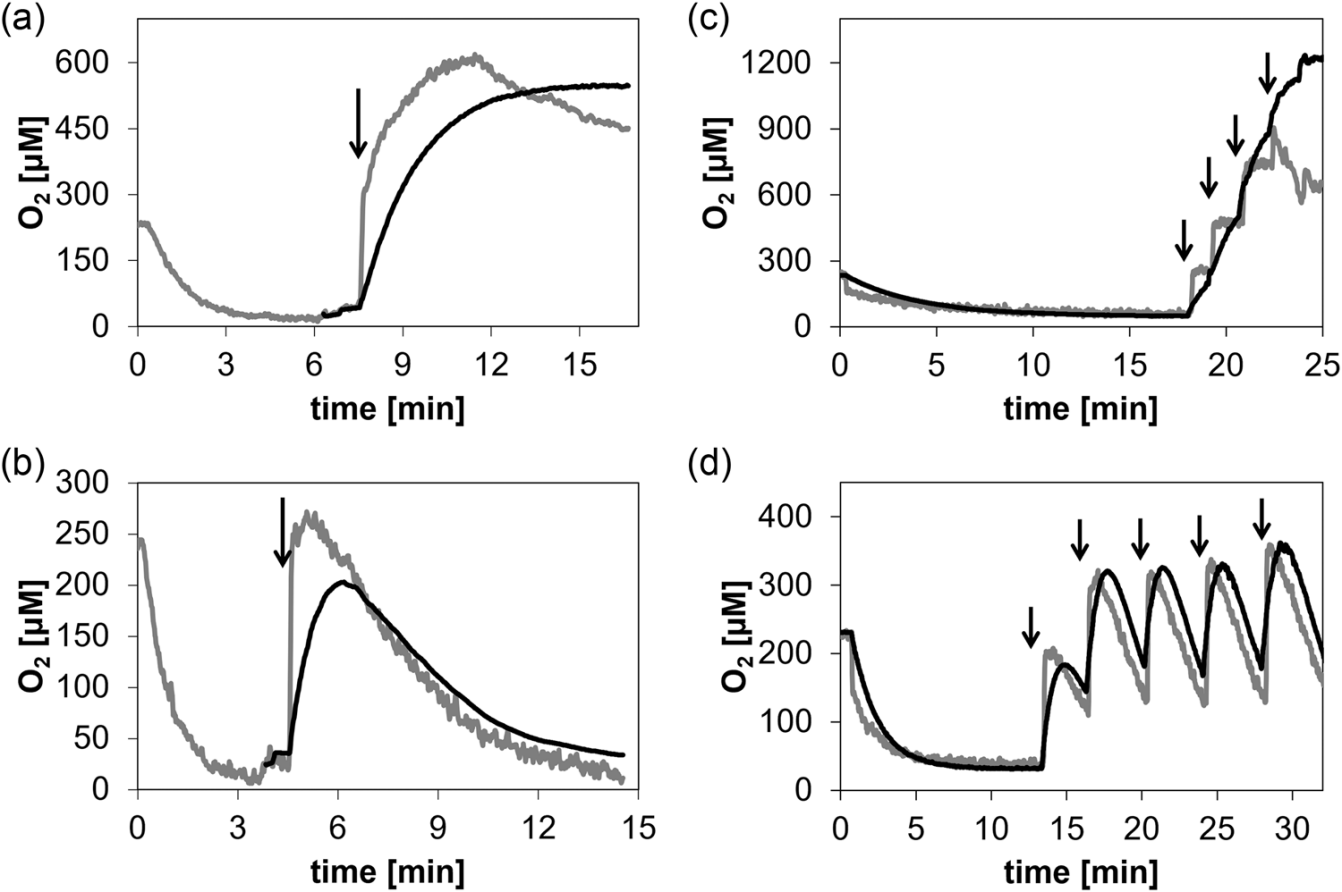
Characterization of internal release and consumption of O_2_. Time courses of internal [O_2_] (gray line, continuous measurement) and external [O_2_] (black line, discontinuous measurement) are shown. (a, b) Relative contributions of diffusion and Z‐DAAO reaction to the dynamics of the internal [O_2_]. (a) Immobilized Z‐CAT (50,000 U/g carrier); (b) co‐immobilized Z‐DAAO (600 U/g carrier) and Z‐CAT (50,000 U/g carrier). Oxygen was depleted in both experiments by bubbling N_2_, and H_2_O_2_ was added afterwards (1.25 mM; arrow). (c, d) Balancing the formation and consumption of internal O_2_. The H_2_O_2_ was intermittently fed (calculated concentration increase, 2.5 mM; arrows) to the enzyme co‐immobilizate (Z‐DAAO: 600 U/g carrier; Z‐CAT: 50,000 U/g carrier;). (c) H_2_O_2_ was added approx. every min. After the fourth addition, bubble formation by excess O_2_ started to interfere with internal O_2_ measurement. Sensor particles floated to the reactor walls at and above the liquid fill level and were thereby removed from the reaction mixture. (d) H_2_O_2_ was added every 3–4 min. No bubble formation was observed and internal O_2_ measurement was possible throughout the experiment. In all panels, the reaction involved 10 mM d‐Met and (co)‐immobilizate was used at 5 mg/ml. For further details, see Section 2.3. DAAO, d‐amino acid oxidase; CAT, catalase.

The co‐immobilized enzyme preparation involved a 174‐fold higher activity loading of Z‐CAT than Z‐DAAO. When the internal [O_2_] is formed from H_2_O_2_ much faster than it is used for oxidation of d‐Met (kinetic simulations described later suggest ∼100‐fold), an observed decrease in the internal [O_2_] can arise from physical diffusion to the surrounding bulk in addition to enzymatic consumption inside the carrier. We therefore performed experiments to assess the relative contributions of diffusion and Z‐DAAO reaction to the dynamics of the internal [O_2_]. After gassing out the O_2_ in bulk liquid (10 mM d‐Met) with N_2_, we added H_2_O_2_ to exactly comparable preparations of immobilized Z‐CAT that lacked or contained additionally immobilized Z‐DAAO, and recorded the corresponding time courses of internal and external [O_2_] as shown in Figure 4a,b. The maximum level of internal O_2_ was considerably higher in the absence (∼600 µM; Figure 4a) compared to the presence of the co‐immobilized Z‐DAAO (∼250 µM; Figure 4b). The maximum [O_2_] reached in the liquid bulk was also much lower when co‐immobilized Z‐DAAO was present. The evidence of Figure 4a,b thus suggests that the dynamics of the internal [O_2_] in co‐immobilized enzyme preparations was affected mostly by the catalytic processes of O_2_ release and consumption inside the carrier, and comparably less by physical diffusion of O_2_ into the liquid bulk. Collectively, therefore, these results demonstrate the function of the co‐immobilized enzymes (internal release of O_2_ substrate from H_2_O_2_ for internal oxidase reaction) exactly as intended. Detail of Figure 4a, that the internal [O_2_] dropped below the external [O_2_] at longer incubation times (≥12 min), can probably be explained by O_2_ diffusion from the particle to the surrounding liquid phase and O_2_ diffusion from the liquid phase to the surrounding air. It can be noted that the O_2_ in the liquid phase reached a stable concentration.

### 3.3 Controlling the addition of H_2_O_2_ with O_2_ measurements inside the solid carrier

The in‐situ supply of O_2_ from periodic pulses of H_2_O_2_ involves H_2_O_2_ amount (concentration and/or volume of the added solution) and time interval of the addition as key operating variables. A simple kinetic model based on mass balance for the coupled reactions of Z‐CAT and Z‐DAAO in solution (Supporting Information: Equations S1–S15) was used to identify promising conditions for the experiment. In particular, overdosing of the H_2_O_2_ was to be avoided. Not only might it harm the immobilized enzymes (Hernandez et al., 2012), but it could also result in uncontrolled O_2_ gas formation. Assuming the standard preparation of co‐immobilized Z‐CAT and Z‐DAAO applied at 5 mg/ml, we determined with kinetic simulation (Supporting Information: Figure S5) that the periodic addition of H_2_O_2_ to a concentration increase of 2.50 mM at intervals of 2–4 min should maintain the O_2_ concentration reasonably within the boundary of O_2_ solubility. At longer intervals of H_2_O_2_ addition (4 min), ∼90% of the H_2_O_2_ added was consumed before the following addition of H_2_O_2_. At shorter intervals (2 min), 30% of the H_2_O_2_ added was still present before the next H_2_O_2_ addition. Therefore, the 4‐min interval of H_2_O_2_ addition was preferred, in particular when considering the possible effects of H_2_O_2_ on enzyme stability and α‐keto acid product decarboxylation (see the discussion in Section 3.5). Optimizing the H_2_O_2_ addition rate is a max‐min problem that requires balance between a high O_2_ concentration for fast production and a low H_2_O_2_ concentration to prevent inactivation and decarboxylation. This was however not pursued in the current study.

Experiments were performed in small‐scale (4 ml) reactions. The H_2_O_2_ was added to a suspension (5 mg/ml) of particles with the co‐immobilized Z‐CAT and Z‐DAAO. The substrate concentration was 10 mM d‐Met. Measurement of the internal [O_2_] served to control the substrate supply from multiple additions of the H_2_O_2_.

In Figure 4c, the H_2_O_2_ was added (Δ[H_2_O_2_] = +2.50 mM) in intervals of ∼1 min, resulting in the fast accumulation of internal and external [O_2_] with each addition. After several additions of H_2_O_2_, as observed visually, gas bubbles were formed in the bulk liquid. This went along with a substantial decrease in the signal‐to‐noise ratio for measurements with the bulk microsensor and the intraparticle sensor (at >900 µM [O_2_]). The deteriorated performance of the intraparticle sensor can probably be explained by gradual removal (flotation) of the labeled particles with the rising gas bubbles. In Figure 4d, we used a slower addition of the same amount of H_2_O_2_ in intervals of 3–4 min consistent with the results of kinetic simulation. Fresh H_2_O_2_ was added only when the internal [O_2_] had decreased to ∼50% of the maximum value from the previous addition. Balance in the formation and consumption of internal O_2_ could thus be shown experimentally.

### 3.4 Assessing enzyme stability in the presence of H_2_O_2_

**Figure 5:**
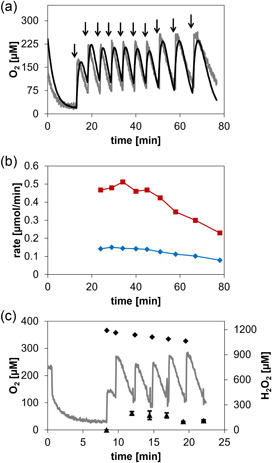
Enzyme stability in the presence of H_2_O_2_. (a) Intermittent H_2_O_2_ feeding (calculated concentration increase of 1.25 mM, indicated by arrows) to the enzyme co‐immobilizate (Z‐DAAO: 600 U/g carrier; Z‐CAT: 50,000 U/g carrier). Internal [O_2_] (gray line, continuous measurement) and external [O_2_] (black line, continuous measurement) were monitored. (b) *r*_total_ (red line and squares) and *r*_con_ (blue line and diamonds) calculated for each H_2_O_2_ addition in panel (a). (c) Monitoring of the H_2_O_2_ consumption by the co‐immobilized enzyme preparation. The H_2_O_2_ was added when the internal [O_2_] had decreased to ~150 µM. The [H_2_O_2_] reached by the addition is indicated by black diamonds. The final [H_2_O_2_] (black triangles) at the end of each H_2_O_2_ addition cycle was determined when the internal [O_2_] (gray line, continuous measurement) had reached the same [O_2_] as before the addition of H_2_O_2_. Error bars show the S.D. from three replicate experiments. In all panels, (co)‐immobilizate was used at 5 mg/ml. For further details, see Section 2.3. DAAO, d‐amino acid oxidase; CAT, catalase.

Considering H_2_O_2_ as a nonspecific oxidant of proteins that causes inactivation broadly among enzymes (Stadtman & Levine, 2003), we sought to analyze how the stability of Z‐CAT and Z‐DAAO was affected by the multiple additions of H_2_O_2_. Figure 5a shows time courses of internal and external [O_2_] in the reaction of the co‐immobilized enzymes when H_2_O_2_ was added ten times, with a Δ[H_2_O_2_] of +1.25 mM in each addition. The intervals between the H_2_O_2_ additions were set based on measurements of the internal [O_2_] to avoid accumulation of O_2_. The data reflect enzyme stability on different parameters as follows. For each H_2_O_2_ addition, the time required for the internal [O_2_] to return to the value before the feed was determined. With this time (Δ*t*), an overall O_2_ consumption rate (*r*_total_) was calculated for each feeding step (Equation 3), considering the stoichiometry of catalase reaction (H_2_O_2_ → H_2_O + ½ O_2_; Figure 1a) and the relevant volume *V* suitably corrected for the volume added.

(3) $$r_{total}=½\Delta \left[H_{2}O_{2}\right]\times V/\Delta t$$

A decrease in *r*_total_ indicates activity loss in Z‐CAT, Z‐DAAO, or both. Figure 5b shows that *r*_total_ was largely constant for the first 5 additions of H_2_O_2_, only to decrease later with each addition to ∼50% of the initial value (~0.5 µmol/min). Results showed earlier (Figure 4a,b) suggest that the rate at which the internal [O_2_] decreases in the carrier is chiefly due to the activity of Z‐DAAO for consuming O_2_. For each H_2_O_2_ addition, therefore, we determined this rate (*r*_con_) from the steepest decline in the internal [O_2_] (Figure 5a). Like *r*_total_, as shown in Figure 5b, the *r*_con_ was almost constant for the first five additions of H_2_O_2_ and dropped to half its initial value during the later additions. These results reveal the partial inactivation of Z‐DAAO upon the repeated addition of H_2_O_2_ and demonstrate the oxidase activity to limit the *r*_total_ of the co‐immobilized enzyme preparation. Note: the shown evidence does not exclude the possibility of the immobilized Z‐CAT to be also inactivated in the process, perhaps even faster than the immobilized Z‐DAAO. It only demonstrates that the used excess of Z‐CAT activity was sufficient for O_2_ release from H_2_O_2_ to not become rate‐determining for *r*_total_. To acquire further evidence in support of this notion, we measured the external concentration of H_2_O_2_ at the points of minimum internal [O_2_] right before fresh H_2_O_2_ was added. Figure 5c shows that a relatively stable, low level of H_2_O_2_ (≤15% of the H_2_O_2_ added) was present over six additions of the H_2_O_2_. This result shows that the immobilized Z‐CAT activity could not have been limiting the conversion of the externally supplied H_2_O_2_.

### 3.5 O_2_ supply via controlled feeding of H_2_O_2_

In a next step of development, we considered supply of H_2_O_2_ in a fully continuous manner. Thus, it should be possible to gain improved control over the [H_2_O_2_] that the enzymes are exposed to during the reaction, with consequent benefits on their operational stability. To better manage the volume change from the H_2_O_2_ feed than was possible in the small‐scale 4 ml working volume, the whole reaction was scaled up to ∼200 ml. The carrier concentration was increased to 15 mg/ml to improve the signal‐to‐noise ratio (∼2.5‐fold compared to 5 mg/ml) for the measurement of the internal [O_2_] in the larger vessel.

**Figure 6:**
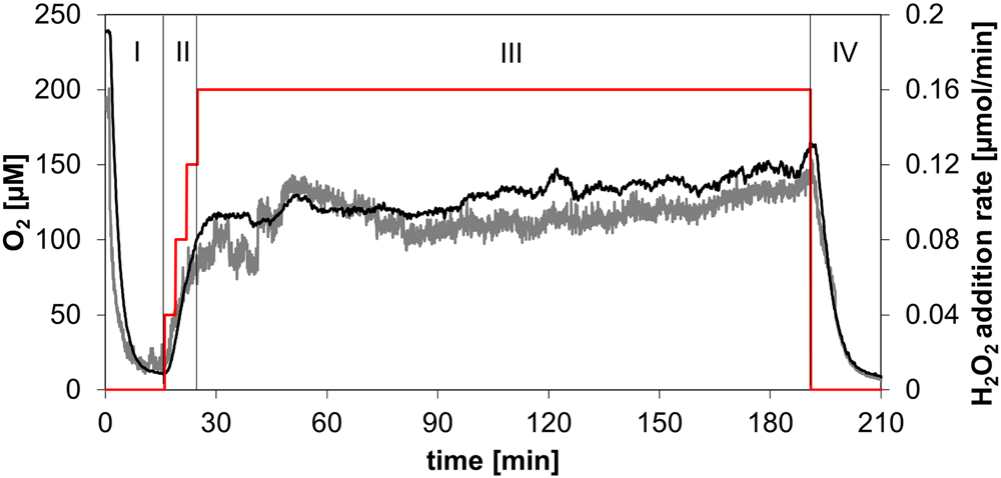
Representative [O_2_] time course for enzymatic reaction under O_2_ supply via controlled feeding of H_2_O_2_. The H_2_O_2_ was added continuously to the enzyme co‐immobilizate (Z‐DAAO: 600 U/g carrier; Z‐CAT: 50,000 U/g carrier; 15.0 mg carrier/ml). Internal [O_2_] (gray line, continuous measurement) and external [O_2_] (black line, continuous measurement) are monitored. The experimental set‐up had four phases: I. d‐Met (200 mM) was added to the reactor and the soluble O_2_ in the reactor was consumed; II. The H_2_O_2_ feeding was started and the flow rate was slowly increased from 0.04 to 0.12 mmol/min every 3 min; III. The H_2_O_2_ was continuously fed at a rate of 0.16 mmol/min; IV. The H_2_O_2_ feed was stopped and the remaining H_2_O_2_ and O_2_ were consumed. For details of the experimental setup used, see Section 2.4. DAAO, d‐amino acid oxidase; CAT, catalase.

Figure 6 shows a typical course of continuous‐feed experiment over 200 min. The H_2_O_2_ feed was switched on after the O_2_ concentration inside the particles and in the bulk had approached low values that appeared to be somewhat steady over time. Starting from 0.04 mmol/min, the rate of H_2_O_2_ addition was ramped up to 0.16 mmol/min (absolute oxidase rate: ∼0.091 mmol/min) in steps of 0.04 mmol/min every 3 min. The absolute oxidase rate is the activity of the immobilized Z‐DAAO measured at air saturation under the same conditions as in the reactor, but in the absence of additionally supplied H_2_O_2_. The final rate of H_2_O_2_ addition (~0.16 mmol/min) was therefore close to double the absolute oxidase rate (∼0.091 mmol/min). Slow start of the H_2_O_2_ addition served the purpose of preventing the excessive formation of O_2_ in the initial reaction phase. In its main part (30–200 min), the reaction was performed at a constant rate of 0.16 mmol/min, resulting in constant and similar O_2_ concentrations (∼130 µM) inside the carrier and in solution. After correction for volume change, the total amount of H_2_O_2_ added was ~27 mmol. At the end of the reaction, the H_2_O_2_ feed was switched off and the rapid consumption of the O_2_ showed that the immobilized Z‐DAAO was still active. Comparison of the absolute O_2_ consumption rates at the start (0.076 mmol/min) and the end of the reaction (0.058 mmol/min) indicated that the Z‐DAAO had retained at least 76% of the initial enzyme activity. Considering that the amount of H_2_O_2_ added through continuous feed was ∼10 times that added batchwise in six steps (Figure 5), the shown evidence suggested a remarkable improvement of Z‐DAAO operational stability as a result of the changed mode of H_2_O_2_ supply.

**Figure 7:**
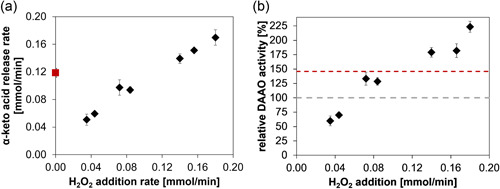
Control of the enzymatic oxidation rate by the H_2_O_2_ feeding rate. Dependence of the α‐keto acid release rate (a; measured with the colorimetric assay) and the enzyme activity (b; expressed from the d‐Met consumption rate) on the H_2_O_2_ feeding rate. The experiment using O_2_ supply by bubble aeration is indicated by a red square in panel (a). Error bars show the S.D. (*N* ≥ 3). The co‐immobilized enzyme preparation (Z‐DAAO: 600 U/g carrier; Z‐CAT: 50,000 U/g carrier) was used at 15.0 mg carrier/ml. In panel (b), the initial rate of reaction at air saturation without external O_2_ supply (Z‐DAAO activity assay) was taken as 100% activity and shown as a gray dashed line. The reaction rate obtained with bubble aeration is shown as a red dashed line. For the experimental setup, see Section 2.4. CAT, catalase; DAAO, d‐amino acid oxidase.

Provided that the H_2_O_2_ formed in the oxidase reaction can be fully used for the recycling of O_2_, the overall oxidase‐catalase transformation involves a theoretical yield of 1 for the product (α‐keto acid) formed on the externally added H_2_O_2_ (Figure 1a). To demonstrate the efficient reaction of the co‐immobilized enzyme preparation, we performed experiments at varied H_2_O_2_ feed rate (0.035–0.185 mmol/min) and determined the corresponding product formation. Samples were analyzed (colorimetric assay) from early reaction times (≤15 min) that largely excluded oxidative decomposition (decarboxylation) of the α‐keto acid promoted by the H_2_O_2_ (Supporting Information: Figure S6). Additional HPLC data confirmed close balance between d‐Met consumed and α‐keto acid released under these conditions (Supporting Information: Figure S7). As shown in Supporting Information: Figure S6, the portion of decarboxylated product was dependent on the H_2_O_2_ addition rate and increased with the reaction time. Samples at 15 min involved a maximum of ∼5% decarboxylation at the highest H_2_O_2_ addition rate. Figure 7a shows the correlation between the H_2_O_2_ feed and α‐keto acid release rates. The relationship was linear (*R* ^2^ = 0.985) with a slope of around unity (0.95 ± 0.05), as expected for a near‐perfectly atom‐economic usage of the H_2_O_2_ supplied. It is noted from Figure 7a that at low H_2_O_2_ addition rates, the rate ratio of product formed and H_2_O_2_ supplied exceeded the theoretical maximum of 1. Limited supply of O_2_ from the surrounding air can plausibly explain the effect. To provide a point of reference, we show in Figure 7a, the α‐keto acid release rate obtained with the enzyme co‐immobilizate under conditions of O_2_ supply by bubble aeration. Unfortunately, the air bubbles interfered with continuous online measurements by the internal and external O_2_ sensors. Lacking suitable monitoring and control in the system used under these conditions, the O_2_ supply from gas (air or pure O_2_) was not further pursued. We also showed that the α‐keto acid production rate was linearly dependent on the loading of co‐immobilized enzyme (range: 5.0–20 mg/ml; data not shown) and the H_2_O_2_ feed rate could be adjusted conveniently to match the demand of the increased conversion rate.

In summary, therefore, these results demonstrate an integrated process concept, together with the associated analytical technology for continuous online measurement of the internal [O_2_], to perform heterogeneously catalyzed O_2_‐dependent conversions under controlled supply of O_2_ substrate from H_2_O_2_ directly inside the solid catalyst (Chapman et al., 2018; Cosgrove et al., 2019; Schneider et al., 2012; Van Hecke et al., 2009; Yoshimoto & Higa, 2014). The proposed approach involves modular elements of bioengineering which enable its flexible application across a variety of enzymatic reactions dependent on O_2_. First, fusion to the Z_basic2_ module promotes facile enzyme co‐immobilization with the Z‐CAT in high yield and effectiveness. Second, the Z‐CAT immobilized on ReliSorb carrier labeled for optical O_2_ sensing represents a well‐characterized “chemical O_2_ generator” module for strictly localized release of O_2_ from H_2_O_2_ (Bolivar et al., 2016; this study) with broad applicability for reaction characterization and process development. Additionally, it can serve as an engineering tool for controlling the reactivity of immobilized O_2_‐dependent enzymes, as shown below.

### 3.6 Reactivity control by the H_2_O_2_ feeding rate

We have shown earlier (Figure 2) that the immobilized Z‐DAAO (with Z‐CAT co‐immobilized) involves a roughly linear profile of the O_2_ consumption rate (i.e., oxidase activity) versus the O_2_ concentration (≤250 µM) in the liquid bulk. It stands to reason, and we have shown this in related studies of Z‐DAAO immobilization (Bolivar et al., 2014, 2015), that the immobilized oxidase activity is also directly dependent on the internally available O_2_ concentration. Results of this study (Figure 6) show that in the co‐immobilized preparation of Z‐CAT and Z‐DAAO the internal [O_2_] is dependent, and hence appears to be tunable, by the H_2_O_2_ feeding rate. The H_2_O_2_ feeding rate is thus identified as an operational parameter to adjust the substrate conversion rate of the Z‐DAAO. We show in Figure 7b that the apparent DAAO activity, calculated from the HPLC‐measured d‐Met consumption rate and expressed relative to the enzyme activity measured in the standard assay, increased roughly 4.5‐fold when the H_2_O_2_ feeding rate was increased from 0.0385 mmol/min to 0.185 mmol/min. The H_2_O_2_‐driven reactions are again compared with reaction of the same co‐immobilized enzyme preparation under conventional O_2_ supply (aeration of the bulk liquid). The highest H_2_O_2_ feeding rate yielded a ∼1.5‐fold enhancement of the d‐Met conversion rate compared to the bubble‐aerated reaction. The rate enhancement with respect to the initial rate measured without external O_2_ supply was ∼2.3‐fold. The internal [O_2_] of ∼130 µM under continuous supply of H_2_O_2_ (Figure 6; H_2_O_2_ feed rate of 0.16 mmol/min) and ≤ 50 µM for the reaction in air‐saturated buffer (Supporting Information: Figure S3) seem to be consistent with the observed factor of rate enhancement. Adjustment of the immobilized oxidase activity by the H_2_O_2_ feeding can provide flexible control of the reactivity of the biocatalytic system. It may also be useful to achieve reaction intensification, particularly in situations when gas–liquid contact at enhanced partial pressure of O_2_ (gassing with pure O_2_, Lindeque & Woodley, 2020; Solé et al., 2019; reaction at elevated pressure, Bolivar et al., 2019; Brummund et al., 2016) is not a preferred option.

## 4 CONCLUSIONS

The co‐immobilization with CAT enables in‐situ supply of O_2_ from H_2_O_2_ to the reaction of solid preparations of immobilized DAAO. Fusion to Z_basic2_ allows for a facile co‐immobilization of the two enzymes (Z‐DAAO and Z‐CAT) in the same porous carrier in suitable activity ratio. Ideally, the O_2_ supply is balanced, providing a suitable intraparticle [O_2_] for a high production rate, while preventing O_2_ overproduction in the bulk surrounding the particle, to avoid loss of control over the reaction. Continuous online measurement of the internal [O_2_] enables controlled feeding of H_2_O_2_. This ensures a programmable O_2_ release rate and avoids enzyme inactivation by excess H_2_O_2_. Enhanced supply of O_2_ to the solid enzyme catalyst can be useful to achieve reaction intensification. The approach demonstrated here for DAAO seems applicable broadly to O_2_‐dependent enzymes. It can facilitate the development of heterogeneous catalysis applications for biocatalytic transformations with O_2_ as the substrate. It presents advance for a general concept of reaction engineering important in applied biocatalysis, not only with O_2_‐dependent enzymes but also with cells (Birmingham et al., 2021; Dong et al., 2018; Tomaszewski et al., 2014; Van Hecke et al., 2011).

## Supporting information

Supporting information.

## Data Availability

The data that support the findings of this study are available from the corresponding author upon reasonable request.
