# Supplementary material for: Monitoring and control of the release of soluble O2 from H2O2 inside porous enzyme carrier for O2 supply to an immobilized d‐amino acid oxidase
Source: Biotechnol Bioeng. 2022 May 16;119(9):2374–87. doi: 10.1002/bit.28130 (PMC9545842; doi:10.1002/bit.28130)
Supplement: Supplementary file 1 — Supporting information. [file BIT-119-2374-s001.docx]

**SUPPORTING INFORMATION**

**Monitoring and control of the release of soluble O_2_ from H_2_O_2_ inside porous enzyme carrier for O_2_ supply to an immobilized D-amino acid oxidase**

Sabine Schelch^a,b^, Juan M. Bolivar^a,b^, and Bernd Nidetzky^a,b,*^

^a^ Austrian Centre of Industrial Biotechnology, Krenngasse 37, A-8010 Graz, Austria

^b^ Institute of Biotechnology and Biochemical Engineering, Graz University of Technology, NAWI Graz, Petersgasse 12, A-8010 Graz, Austria

*Corresponding author: Bernd Nidetzky, e-mail: bernd.nidetzky@tugraz.at, phone: +43 316 873 8400

**SUPPORTING TABLES**

Table S1. Yields and effectiveness factors (η) of enzyme immobilizate and co-immobilizate in all steps of a consecutive co-immobilization, starting with either Z-DAAO or Z-CAT for the first round of immobilization. Final enzyme loading: 600 U Z-DAAO/g carrier and 50,000 U Z-CAT/g carrier. Labeling was 2.5 mg Ru(dpp)_3_ dye/g carrier.

|  | | | Z-DAAO immobilized first | Z-CAT immobilized first |
| --- | --- | --- | --- | --- |
| Immobilization  First enzyme | Yield  [%] | Z-DAAO | 98^a^ | - |
|  |  | Z-CAT | - | 96^b^ |
|  | η  [%] | Z-DAAO | 8.5^c^ | - |
|  |  | Z-CAT | - | 7^b^ |
| Immobilization  Second enzyme  (Co-immobilization) | Yield  [%] | Z-DAAO | - | 97^a^ |
|  |  | Z-CAT | 97^b^ | - |
|  | η  [%] | Z-DAAO | 5.5^c^ | 4^c^ |
|  |  | Z-CAT | 3^b^ | 3.5^b^ |
| Labeling with Ru(dpp)_3_ dye | Yield [%] | | 100 | 100 |
|  | η  [%] | Z-DAAO | 3^c^ | 2.5^c^ |
|  |  | Z-CAT | 4.5^b^ | 3^b^ |

^a^ Coupled peroxidase assay
^b^ H_2_O_2_ assay
^c^ O_2_ consumption recorded by external oxygen sensor
Analytics are described in the main text (2.1) and immobilization parameters are defined in 2.2 of the main text.

**SUPPORTING FIGURES**


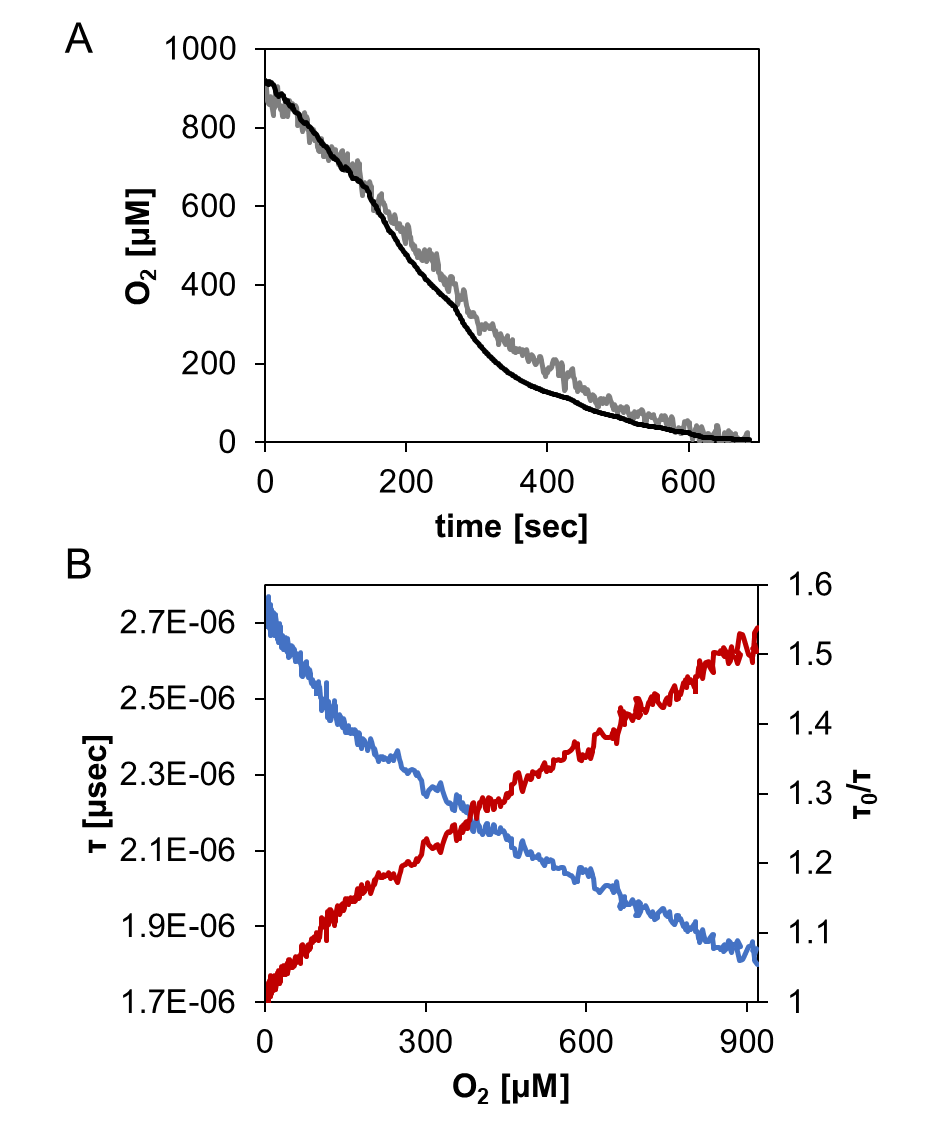

Figure S1: Analysis of the O_2_ concentration dependence of the luminescence lifetime, τ, of the Ru(dpp)_3_-labeled co-immobilizate of Z-DAAO (600 U/g carrier) and Z-CAT (50,000 U/ g carrier). (A) After O_2_ supply with pure O_2_ gas, the soluble [O_2_] was decreased again with glucose oxidase (5 U/ml) and glucose (100 mM); the graph shows the consumption of [O_2_] by the glucose oxidase reaction. The glucose oxidase was added at time zero of the experiment. External O_2_ (black line, continuous measurement) and internal O_2_ (grey line, continuous measurement) are shown. (B) Recorded τ values (blue) or τ ratios (red) for Ru(dpp)_3_ over a range between 0 and 920 µM O_2_.


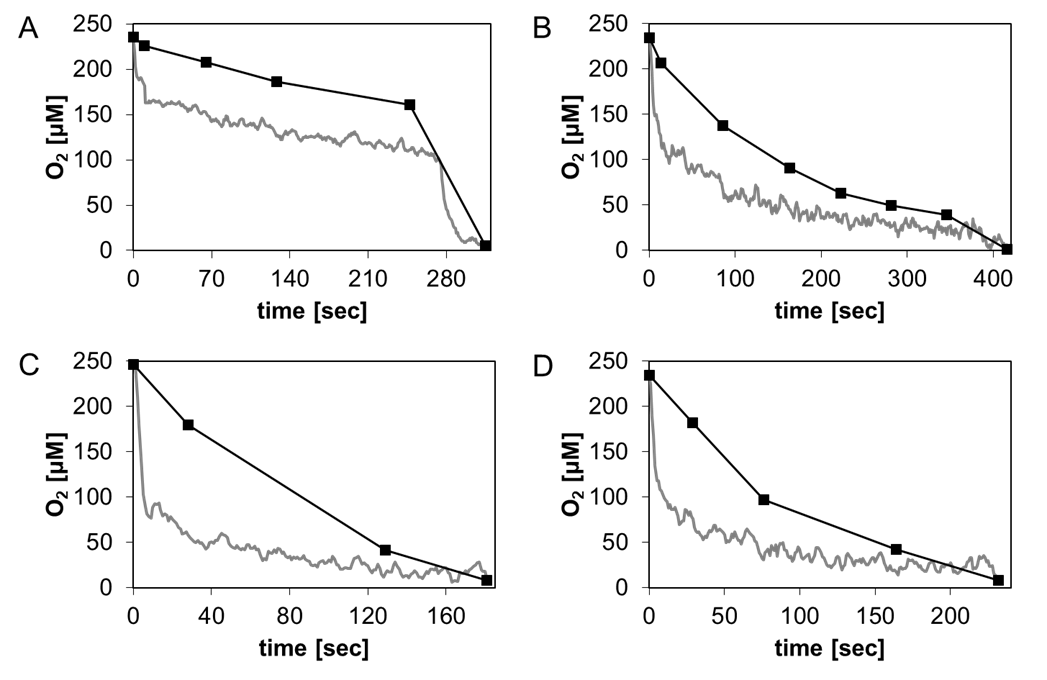


Figure S2: Measurement of O_2_ consumption after addition of D-Met (Δ[D-Met] = +10 mM) to immobilized Z-DAAO of various enzyme loadings: (A) 200 U/g carrier; (B) 400 U/g carrier; (C) 600 U/g carrier; (D) 800 U/g carrier. External O_2_ (black line with squares, point measurement at each square) and internal O_2_ (grey line, continuous measurement) are shown. No additional O_2_ was supplied to the reactor before, or during, the experiments.


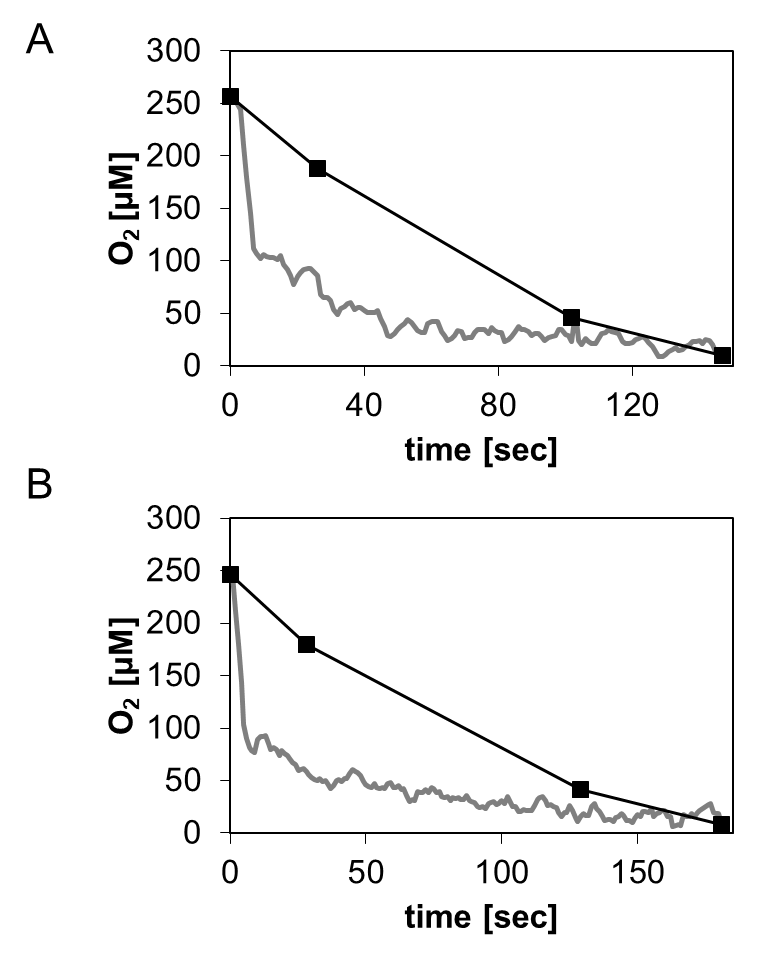


Figure S3: Measurement of O_2_ consumption after addition of D-Met (Δ[D-Met] = +10 mM) to (A) immobilized Z-DAAO (600 U/g carrier) and (B) co-immobilized Z-DAAO (600 U/g carrier) and Z-CAT (10,000 U/g carrier). External O_2_ (black line with squares, point measurement at each square) and internal O_2_ (grey line, continuous measurement) are shown.


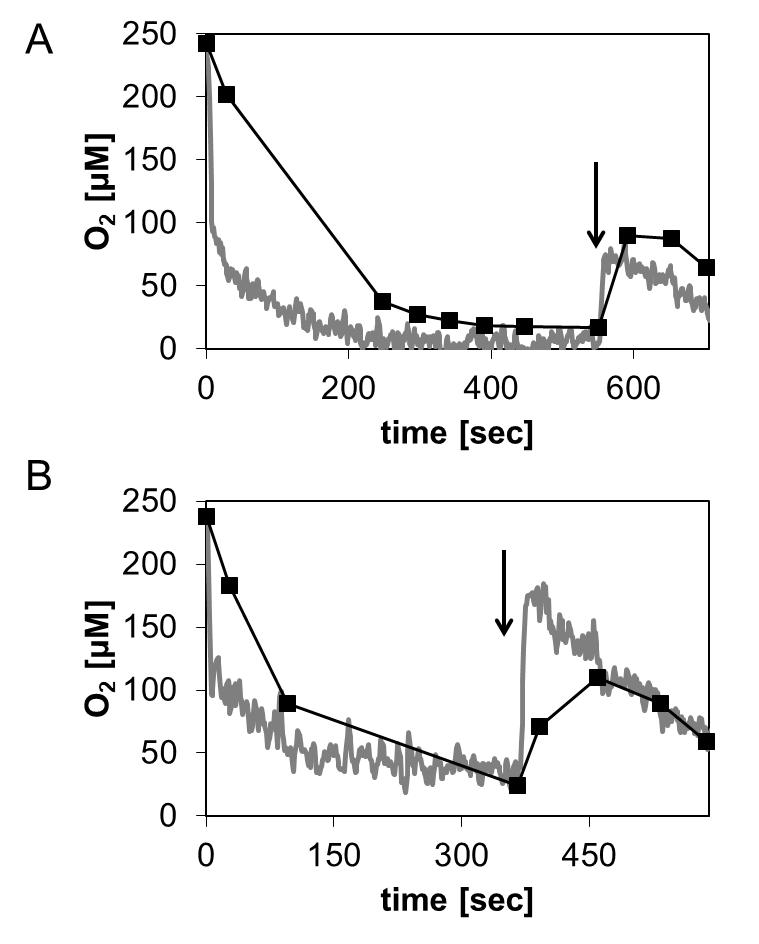


Figure S4: Measurement of oxygen consumption after addition of D-Met (Δ[D-Met] = +10 mM) and addition of H_2_O_2_ (Δ[H_2_O_2_] = +2.5 mM; arrow) to (A) co-immobilized Z-DAAO (600 U/g carrier) and Z-CAT (10,000 U/g carrier) and (B) co-immobilized Z-DAAO (600 U/g carrier) and Z-CAT (50,000 U/g carrier). External O_2_ (black line with squares, point measurement at each square) and internal O_2_ (grey line, continuous measurement) are shown.


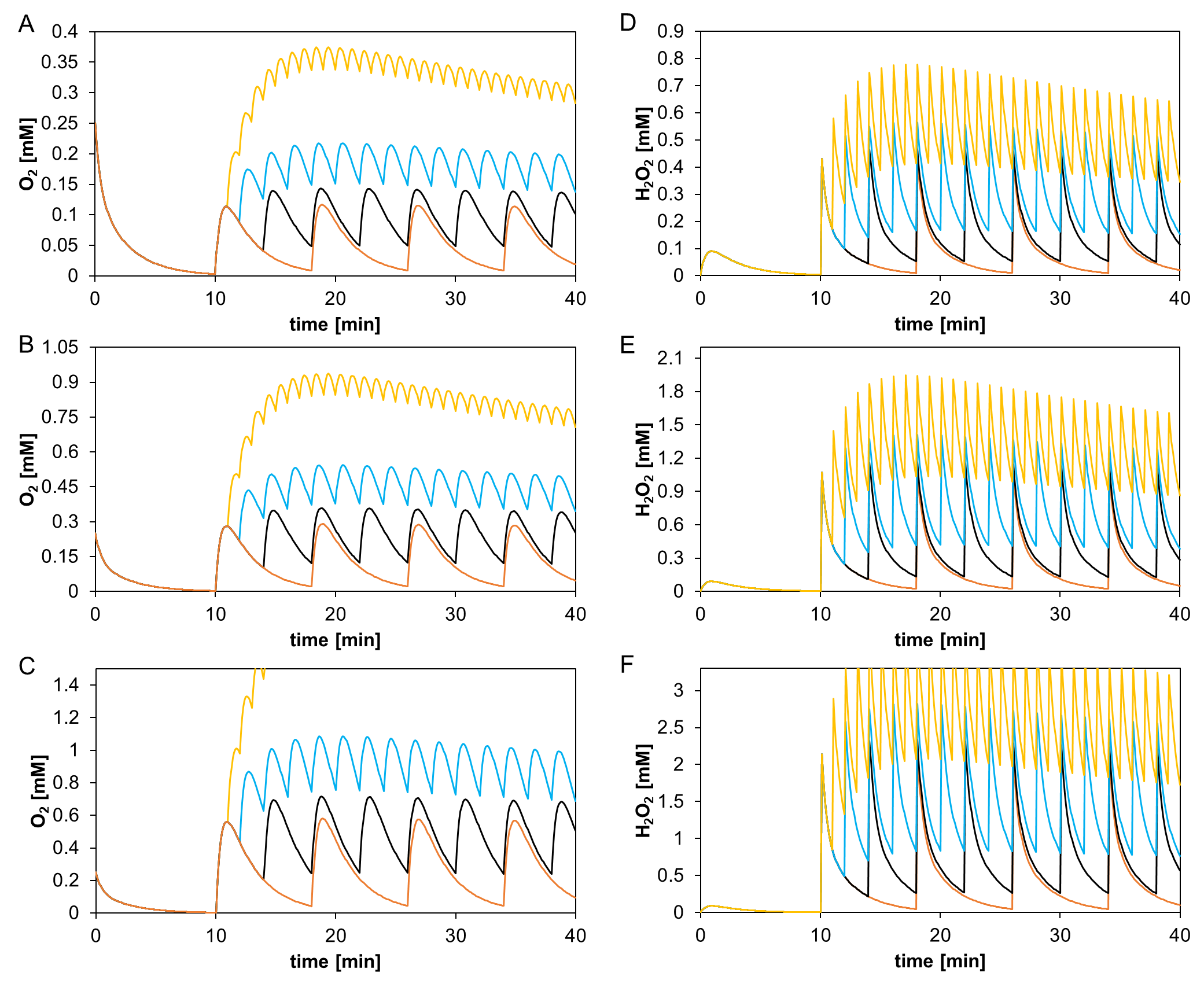


Figure S5: Simulation of time courses of O_2_ (panels A-C) and H_2_O_2_ (panels D-F) in the H_2_O_2_-driven reaction of Z-DAAO and Z-CAT. The simulated reactions involved the repeated addition of 50 µl (Vol_add) of H_2_O_2_ stock solution at the indicated times. The initial volume of the reaction was 4 ml, as in the experiments. The stock concentration was varied so that the same Vol_add achieved a change in the H_2_O_2_ concentration (Δ[H_2_O_2_]) in the reaction of 0.5 mM (panels A, D), 1.25 mM (panels B, E) and 2.5 mM (panels C, F). The results were obtained by applying the (numerically integrated) kinetic model described in section S1 of this Supporting Information. The time intervals of addition (Time_ad) of H_2_O_2_ were simulated to vary between 1 and 8 min, as indicated by color of the curves. The results show how the H_2_O_2_ dosage (ΔH_2_O_2_/Time_ad) defines a pseudo-steady state of [O_2_] that is proportional to ΔH_2_O_2_/Time_ad. A pseudo-steady state of air saturation is reached when ΔH_2_O_2_/Time_ad approaches the volumetric activity (based on air-saturated conditions).


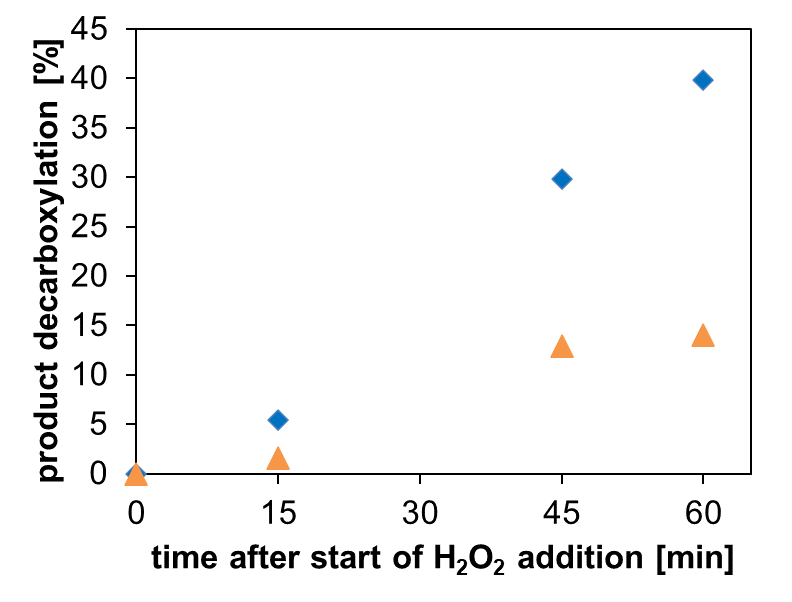

Figure S6: The α-ketoacid product degradation dependent on the H_2_O_2_ addition rate. The degradation was calculated from the difference of D-Met consumption and α-keto acid production, measured with HPLC (Figure S7) over a reaction time of 60 min. The H_2_O_2_ addition rate was varied (orange triangle, 0.08 mmol/min; blue diamond, 0.18 mmol/min) in fed-batch mode for the reactor operated with co-immobilized Z-DAAO (600 U/g carrier) and Z-CAT (50,000 U/g carrier).


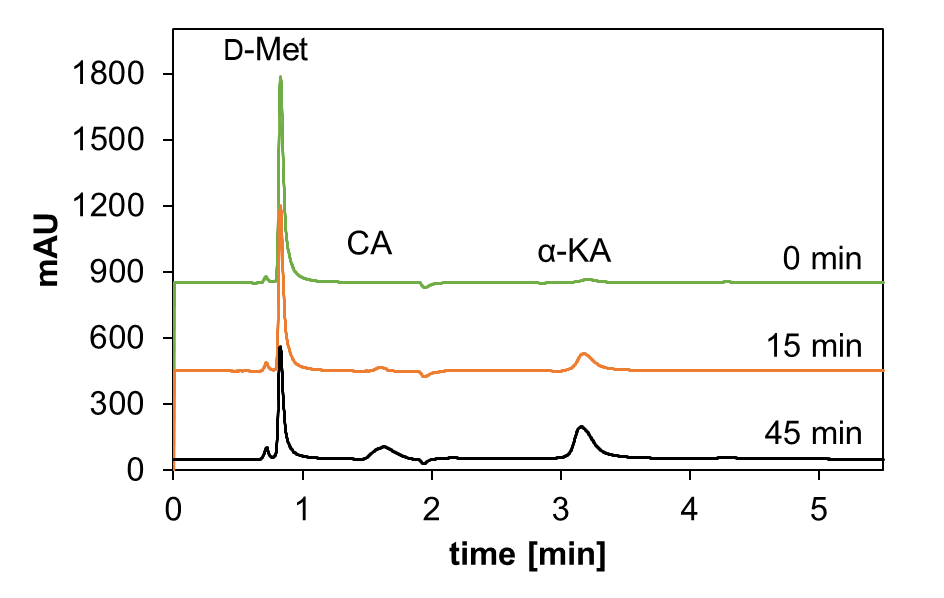


Figure S7: HPLC analysis of D-Met consumption and formation of α-keto acid and decarboxylated products. Chromatograms of samples taken from the reactor with the co-immobilizate at the indicated times after starting H_2_O_2_ addition. α-KA (α-keto acid; α-keto-γ-(methylthio)-butyric acid); CA (the likely decarboxylation product of the α-KA, β-(methylthio)-propionic acid).

**SUPPORTING METHODS**

**S1 Kinetic modeling of substrate consumption and product formation in a coupled reaction of Z-CAT and Z-DAAO upon addition of H_2_O_2_**

Kinetic model for coupled enzymatic reaction in solution was developed. Note: the model does not describe reaction-diffusion for the co-immobilized enzyme system. The aim of this model was to support experiments in which H_2_O_2_ was added intermittently. The experiment requires decision of how much H_2_O_2_ to add in which time interval. The model helps to identify suitable conditions for experiment.

O_2_ (Equation S1) and product formation (Equation S2), D-Met consumption (Equation S3) and H_2_O_2_ balance (Equation S4) were simulated. Berkeley Madonna^TM^ (Version 10.2.8 ) was used for numerical solution of the model.

$\frac{d \mathrm{nO}_{2}}{dt}=\left( -r_{ox}+0.5 r_{cat} \right) Vol$ (S1)

$\frac{d nketo acid}{dt}=r_{ox} Vol$ (S2)

$\frac{d \mathrm{nMet}}{dt}=r_{ox}$ *Vol* (S3)

$\frac{d nH_{2}O_{2}}{dt}=\left( r_{ox}-r_{cat} \right)Vol+$ ${H_{2}O}_{2}\_pulse$ (S4)
*t* is time, nO_2_ is the number of O_2_ moles in the reactor volume (*Vol*)*,* nketoacid is the number of keto acid moles in *Vol*, nMet is the number of D-Met moles in *Vol*, nH_2_O_2_ is the number of H_2_O_2_ moles in *Vol*. *r*_ox_ and *r*_cat_ are the reaction rates of oxidase and catalase, respectively.

The concentration of the different species was calculated according to Equation S5 - S8.

$\left[ O_{2} \right]=\frac{nO_{2}}{Vol}$ (S5)

$\left[ \mathrm{ketoacid} \right]=\frac{\mathrm{nketoacid}}{Vol}$ (S6)

$\left[ \mathrm{Met} \right]=\frac{\mathrm{nMet}}{Vol}$ (S7)

$\left[ {H_{2}O}_{2} \right]=\frac{n{H_{2}O}_{2}}{Vol}$ (S8)
Where *Vol* is the reaction volume calculated according to Equation S9.

$Vol=Vol\_ini+Vol\_pulse$ (S9)

*Vol*_ini is the initial volume and *Vol*_pulse is the volume added in each pulse addition.

To describe the kinetics of DAAO and catalase reactions, Equation S10 and Equation S11 were respectively used. *r_ox_* (Equation S10) is the rate of oxygen consumption catalyzed by the Z-DAAO. For the kinetics of oxidation catalyzed by Z-DAAO (*r*_ox_), it is assumed, in agreement with the experimental conditions (see Figure 2 of main text), that there is a large excess of the D-amino acid substrate over the limiting O_2_ and that there is a first order kinetic dependency between rate and [O_2_]. A value of *k*_o2_ = 1.2 min^-1^, corresponding to an apparent oxidase volumetric activity of 0.3 mM min^-1^, was used for the analysis. For the kinetics of H_2_O_2_ conversion by catalase (*r*_cat_), a first order kinetic model similar to that for Z-DAAO was used. Literature (Switala and Loewen, 2002) showing very high *K*_m_ value (154 mM) for H_2_O_2_ of the *B. pertussis* catalase used justify the rate model of Equation S11. A value of *k*_H2O2_ = 1.2 min^-1^ was assumed. This corresponds to a 100-fold larger activity of catalase compared to oxidase when measured at standard conditions (note: [H_2_O_2_] = 20 mM; [O_2_] = 200 µM at air saturation).

$r_{\mathrm{ox}}= k_{o2} [O_{2}]$ (S10)

$r_{\mathrm{cat}}=k_{H2O2} [H_{2}O_{2}]$ (S11)

The pulsed addition of H_2_O_2_ solution was considered in the balance for H_2_O_2_ (Equation S4) and *Vol* (Equation S9) by including the terms H_2_O_2__pulse and *Vol*_pulse. Here, H_2_O_2__pulse refers to instantaneous molar addition of H_2_O_2_. This pulse was simulated by using the function *Conveyor* (Equation S12) and *Pulse* (Equation S13) from Berkeley *Madonna^TM^ (Version 10.2.8).*

${H_{2}O}_{2}\_pulse=conveyor\left( input, DT \right)/DT$ (S12)

$input=pulse (Vol\_ad H2O2\_stock, Time\_start, Time\_ad)$ (S13)

DT (10^-4^) is the integration time (infinitesimal time of the duration of the H_2_O_2_ addition). A transition time of DT was considered in the *Conveyor* function to include the effect of the instantaneous addition in coherency with the integration step of the numerical solution of the model. *Vol*_ad is the addition volume of H_2_O_2_, H_2_O_2__stock is the concentration of the stock of H_2_O_2_, Time_start is the time where pulse addition of H_2_O_2_ starts, and Time_ad is the time interval of pulse addition.

For the volume balance, the pulse addition of small volumes (*Vol*_ad) was also simulated by using the function *Conveyor* (Equation S14) and *Pulse* (Equation S15) from Berkeley *Madonna^TM^ (Version 10.2.8).* DT is not needed since the *Vol* definition does not involve a differential equation. A transition time of the total duration of the simulated experiment was implemented into the *Conveyor* function to allow for the accumulation of *Vol*.

$Vol\_pulse=conveyor\left( input2, 40 \right)$ (S14)

$input2=pulse (Vol\_ad,Time\_start, Time\_ad)$ (S15)

**REFERENCES**

Switala, J., Loewen, P.C., 2002. Diversity of properties among catalases. Arch. Biochem. Biophys. 401, 145–154. https://doi.org/10.1016/S0003-9861(02)00049-8
